# Drones Detection Using a Fusion of RF and Acoustic Features and Deep Neural Networks

**DOI:** 10.3390/s24082427

**Published:** 2024-04-10

**Authors:** Alan Frid, Yehuda Ben-Shimol, Erez Manor, Shlomo Greenberg

**Affiliations:** 1 School of Electrical and Computer Engineering, Ben Gurion University, Beer Sheva 8410501, Israel; alanfr@post.bgu.ac.il (A.F.); benshimo@bgu.ac.il (Y.B.-S.); 2 Department of Computer Science, Sami Shamoon College of Engineering, Beer Sheva 8410501, Israel; erezmano@post.bgu.ac.il

**Keywords:** drones detection, machine learning, data fusion, acoustic features, radio frequency, wavelets transform

## Abstract

The use of drones has recently gained popularity in a diverse range of applications, such as aerial photography, agriculture, search and rescue operations, the entertainment industry, and more. However, misuse of drone technology can potentially lead to military threats, terrorist acts, as well as privacy and safety breaches. This emphasizes the need for effective and fast remote detection of potentially threatening drones. In this study, we propose a novel approach for automatic drone detection utilizing the usage of both radio frequency communication signals and acoustic signals derived from UAV rotor sounds. In particular, we propose the use of classical and deep machine-learning techniques and the fusion of RF and acoustic features for efficient and accurate drone classification. Distinct types of ML-based classifiers have been examined, including CNN- and RNN-based networks and the classical SVM method. The proposed approach has been evaluated with both frequency and audio features using common drone datasets, demonstrating better accuracy than existing state-of-the-art methods, especially in low SNR scenarios. The results presented in this paper show a classification accuracy of approximately 91% at an SNR ratio of −10 dB using the LSTM network and fused features.

## 1. Introduction

The widespread use of unmanned aerial vehicles (UAVs) during the last decade has led to a growing demand for the development of systems for monitoring and identifying air traffic in low-altitude airspace. Recently, the exploitation of UAV technology has received considerable attention in many applications, such as data acquisition, aerial photography, agriculture, search and rescue operations, and the entertainment industry [1,2,3,4,5]. Along with benefiting from the proposed new technology, the threat posed by drones also increases. Protection against drones and unmanned aerial vehicles requires the development of systems to detect and neutralize the threatening drone. The growing popularity of the use of drones, together with the threatening potential of their misuse, especially from a security and military point of view, sharpens the need for the automatic detection and location of drone swarms in the airspace [6,7,8].

There are four main drone detection methods in use today: (a) RF-based detection [9,10,11,12], which is a method that uses different radio frequency (RF) signatures of the drone’s transmitters based on spectral analysis; (b) acoustic-based detection [13,14,15,16], which uses the drone’s sound and the unique drone’s acoustic features; (c) image-based detection [17,18,19,20], which is a visual detection of a drone within a video frame; and (d) radar-based detection [21,22,23,24], which is based on utilizing advanced radar systems and techniques. S. Singha and B. Aydin [25] proposed an image-based method using a convolutional neural network (CNN) for drone classification, demonstrating an accuracy of 95%. Similar works that use image-based drone classification and CNN demonstrate an average accuracy of 80–90% [17,18,19]. R. Fu et al. [26] presented drone classification at millimeter-wave radars using deep learning techniques. The authors used a long short-term memory (LSTM) network and an adaptive learning rate optimizing (ALRO) model to train the LSTM. The proposed LSTM-ALRO model can work well under a highly uncertain and dynamic environment. They achieved an accuracy of 99.88%. Similar works based on the radar for drone classification in different radar systems (1–4 GHz) can achieve a high accuracy of 95–100% using machine learning methods [22,23,24]. Currently, the leading drone detection techniques are based on either RF or acoustic signals [15,27,28,29,30,31,32,33,34,35,36,37]. Therefore, the following literature review focuses on related work that is based on these two approaches.

The common use of neural networks for drone detection has been proven to be an efficient and successful approach compared to the classic classification methods. Therefore, in this study, we adopt deep machine learning-based approaches. In addition, since the leading drone detection techniques are based on either RF signature or acoustics signals, this research focuses on relevant features that can be extracted from such signals. Features derived from a time-frequency domain, such as MFCC/GTCC and Wavelets, are essential for using recurrent neural networks (LSTM and GRU) that require sequences as their input.

This research proposes a novel approach based on a fusion of both RF signatures and acoustic features. We propose the use of both classical and deep machine-learning techniques, as well as the fusion of RF and acoustic features for efficient and accurate drone classification. Distinct types of machine learning (ML)-based classifiers have been examined including Deep Neural Network (DNN)- and Recurrent Neural Network (RNN)-based networks, as well as the classical SVM method.

The main contributions of this paper are:

- A fusion approach merging RF and Audio features to improve drone classification.
- Evaluating the efficiency of various features in the time-frequency domain.
- A new efficient RNN-based approach applied to multi-class drone classification.
- The proposed method outperforms existing classifiers in a noisy environment.

The rest of this paper is organized as follows: Section 2 presents an overview of related work. Section 3 describes the proposed approach, including the RF and Audio dataset, together with their various features, and the different classifiers used. Section 4 presents the experimental results, and Section 5 contains a summary of the paper.

## 2. Related Work

The leading drone detection techniques are based on either RF or acoustic signals, and therefore the following literature review focuses on related work that is based on these two approaches. Section 2.1 describes various RF-based drone classification approaches, while Section 2.2 presents Audio-based drone classification-related works.

### 2.1. RF-Based Classification

RF-based drone classification leverages the unique RF signatures of drone RF transmitters, in order to distinguish between different drone types. This can be achieved using spectral-based techniques, such as power spectral density (PSD), the short-time Fourier transform (STFT), wavelet-based transforms, mel frequency cepstral coefficients (MFCCs) and GammaTone cepstral coefficients (GTCC). While the latter two techniques are typically employed in audio signal processing, previous studies have demonstrated their efficacy for drone classification.

Al-Sa’d et al. [27] created the DroneRF dataset by collecting, processing, and logging raw RF signals from various drones in four different flight modes. They used a DNN classifier to detect, identify, and determine the flight mode of drones, using the PSD as the feature vector. The classification task was divided into three parts: (1) drone presence detection (two classes); (2) drone presence and type identification (four classes); and (3) drone presence, type identification, and flight mode determination (ten classes). The DNN classifier achieved accuracy rates of 99.7%, 84.5%, and 46.8% for the three tasks, respectively, indicating that accuracy decreases significantly as the number of classes increases.

Allahham et al. [28] present a one-dimensional Convolutional Neural Network (CNN) model, and show great improvement with respect to previous work, using the same DroneRF dataset. The first test (i.e., two classes) achieves near-perfect accuracy, 94.6% for four classes and 87.4% for ten classes.

Medaiyese et al. [29] propose a machine learning-based system for drone detection and identification, which uses low band RF signals from drone to flight-controller communication. Three machine learning models were developed using the XGBoost algorithm to detect and identify the presence of a drone, the type of drone and the operational mode of the detected drone. The XGBoost models achieve average accuracies (using cross validation techniques) of 99.96%, 90.73% and 70.09%, for two, four and ten classes, respectively.

Kılıç et al. [31] demonstrate the similarity between RF and audio signals in terms of time- and frequency-dependent characteristics. The proposed approach utilizes well-established spectral-based audio features like PSD, MFCC, and linear frequency cepstrum coefficients (LFCC) within a Support Vector Machine (SVM)-based machine learning framework. Parameters for feature extraction, including the number of cepstral coefficients, filter bank frequency range, and center frequencies, are optimized for drone RF signals. The study also explores the effectiveness of employing RF signal frequency bands either individually or collectively to achieve optimal performance. The evaluation employs the DroneRF dataset with 0.25 [Sec] samples, processed as single segments, resulting in reduced computational load and improved classification performance. The experimental results show exceptional outcomes: 100% accuracy for drone presence detection (two classes), and 98.67% and 95.15% accuracy for drone type detection (four classes) and operational mode detection (ten classes) using LFCC-based features.

Nguyen et al. [10] introduced “Matthan”, a system for detecting drones by analyzing unique Wi-Fi signal patterns caused by drone body vibrations and shifts. The joint detector combines a frequency-based approach (50–220 Hz range) for identifying body vibration with a wavelet-based method that captures sudden body shifts through temporal RF signal analysis. In various scenarios, including non-drone cases (e.g., Wi-Fi-equipped car, walking user with a smartphone), Matthan achieves over 90% accuracy. Matthan’s effectiveness was consistent across seven drone types and three environments.

Medaiyese et al. [38] propose a semi-supervised technique to detect UAVs by analyzing RF signal fingerprints between a UAV and its flight-controller via Bluetooth and Wi-Fi. They decompose RF signals using a two-level wavelet packet transform to estimate coefficient variance, forming a feature set. The authors use a local outlier factor model for UAV detection based on coefficient variances from Wi-Fi and Bluetooth signals. Accuracy reached 96.7% and 86% for RF-based UAV detection at signal-to-noise ratios (SNR) of 30 dB and 10 dB, respectively. This method has broader applications, including identifying rogue RF devices in various environments. Medaiyese et al. [39] compare a Wi-Fi and Bluetooth-interference resilient drone detection and identification system (DDI), using RF. They employ machine learning, including a pre-trained CNN, named “Squeeze Net”, to classify signals. The study evaluates varying group-device classes (three or ten) and signal states (transient or steady-state), achieving 98.9% accuracy at 10 dB with RF scattergrams.

Ashush et al. [30] introduced the “XBee” dataset, featuring 10 classes of XBee transceivers. They demonstrated high accuracy in distinguishing classes by leveraging minor built-in differences. Employing wavelets and unsupervised methods such as K-Nearest Neighbors (KNN) together with t-Distributed Stochastic Neighbor Embedding (t-SNE) for dimension reduction, they attained a 99% detection rate for SNR up to −8 dB using features embedded by Wavelet Scattering Transform.

### 2.2. Audio-Based Classification

Bernardini et al. [15] employed MFCC and SVM to detect drones amidst clutter noise such as crowds, nature, and trains, achieving 97.4% accuracy. Jeon et al. [32] introduce a binary model using audio data for drone detection. Using a Recurrent Neural Network (RNN), they achieved 81% accuracy, outperforming the CNN and the Gaussian Mixed Model. Al-Emadi et al. [33] validated drone detection and identification using CNN, RNN, and Convolutional-RNN (CRNN) in real scenarios. Using the Short-time Fourier Transform (STFT), they achieved 57.16%, 92.94%, and 92.22% accuracies with RNN, CNN, and CRNN, respectively, while also providing a dataset that we use in this research. Anwar et al. [34] proposed an ML framework using MFCC and Linear Predictive Cepstral Coefficients for feature extraction, achieving 96.7% accuracy for drone detection with SVM cubic kernel. Cabrera-Ponce et al. [35] proposed a UAV-mounted microphone array with a CNN based on the Inception V3 network, detecting UAV presence with 90.1% accuracy. Salman et al. [36] analyzed five audio features, and identified the GammaTone Cepstral Coefficients (GTCC) as efficient for drone detection, achieving 99.9% accuracy with a Gaussian SVM kernel. Katta et al. [37] benchmarked DNN, CNN, LSTM, and Convolutional-LSTM (CLSTM), achieving 98.52%, 98.6%, 98.11%, and 98.6% accuracy, respectively.

## 3. Proposed Approach

This section presents the research methodology and the proposed approach. We suggest using machine learning- and DNN-based approaches for drone characterization and detection based on RF signal analysis and acoustic features. The proposed method utilizes different radio frequency fingerprints (RFF), based on the fact that each transmitter has a unique RFF that arises from imperfections in the analog components during the manufacturing process (Brik et al. [40]). In particular, we suggest using a fusion of both RF and acoustic features to improve existing drone detection classification methods.

Most of the published studies related to single drone detection suggest using either the drone radio frequency signature, or the acoustic features derived from the UAV rotor sounds. In contrast, we propose to utilize both the RF and acoustic features. To the best of our knowledge, this is the first work that applies feature fusion for drone detection using machine learning approaches. The main advantage of the proposed approach is that it is immune to noise, and it admits a significant improvement in detection and classification in a noisy environment with low SNR levels.

RF-based drone classification leverages the uniqueness of drone RF transmitter signature to discriminate between different drone types. We examine various spectral features, such as power spectral density (PSD), the short-time Fourier transform (STFT), MFCC, and wavelet-based transforms. While the latter two techniques are typically employed in audio signal processing, previous studies have demonstrated their efficiency in drone classification using frequency features. We investigated the effectiveness of state-of-the-art ML-based classification methods, including DNN, CNN, LSTM, and the classical SVM method, for drone detection.

The proposed fusion-based method is focused on Recurrent Neural Networks (RNNs), which are well-suited for modeling sequential data. RNNs can learn long-term dependencies in sequential data, and hence guarantee not only robust, but more accurate classifications. The performance evaluation of the proposed fusion-based method is carried out using common RF and Audio drone datasets [27,30,33].

**Figure 1:**
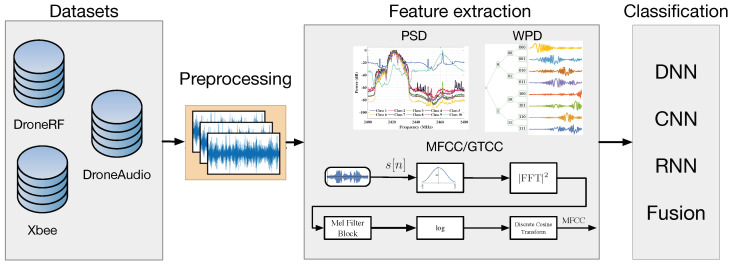
The proposed method flow diagram.

The proposed method includes the following main phases: (a) RF and Audio datasets generation, (b) preprocessing and various feature extraction, (c) feature fusion using an RNN-based approach, and (d) applying ML-based methods for drone classification. Figure 1 depicts the main stages of the proposed approach, including data acquisition, preprocessing, feature extraction, RNN-based feature fusion, and classification, as described in the following sections.

### 3.1. Datasets

This section describes the RF and Acoustic datasets used to evaluate the proposed approach. In our previous work [30], we developed a self-built RF-dataset, based on ZigBee, as described in Section 3.1.1. The ZigBee protocol has been applied to drone-to-drone communication in a physical scenario of swarm drones [41,42] supporting a large number of nodes. In addition to the self-built XBee dataset, we used several common datasets published in the literature, described in Section 3.1.2 and Section 3.1.3.

#### 3.1.1. XBee Self-Built RF Dataset [30]

The Xbee dataset includes 10 XBee ZB S2C-based transmitters using ZigBee communication, where one serves as a coordinator device. All the XBee modules are configured with the same properties. We used a GNU radio platform with SDR to acquire the RF signals [43].

#### 3.1.2. DroneRF Dataset

We adapted the RF database provided by Al-Sa’d et al. [27]. This database contains raw RF signals acquired from three drones operating under different flight modes such as: “off”, “on and connected”, “hovering”, “flying”, and “video recording” [27]. The following type of drones have been used to build the database: *Parrot Bebop*, *Parrot AR*, and *DJI Phantom*, where all drones use Wi-Fi operated at 2.4 GHz. The database contains RF background activities when drones are absent, and RF drone activities when drones are present.

#### 3.1.3. Drone Audio Dataset

Due to the lack of public drone audio datasets available for drone detection, we have adapted the dataset presented in [33] and the unique SPCup19 dataset [44].

The dataset acquired in [33] was collected from two commercial drones, the Bebop and the Mambo drones manufactured by Parrot. To acquire the drone sounds, audio clips of the sound generated by the drone’s propellers while flying and hovering in a quiet indoor environment have been recorded. The dataset includes 1300 audio clips of drone sounds, with a total sound clip of about 11 min per drone. A portion of the dataset includes also pure noise and silence. The SPCup19 ego-noise database [44] has been generated from drone engine-noise data, within the 2019 IEEE Signal Processing Cup by the ten participating teams gathering a unique database of drone-embedded recordings.

The drone audio dataset used in this work consists of a recording of nine different drone propeller sounds and an interference class. Table 1 shows the drone audio dataset, which is composed of seven drones from [44] and three classes from [33].

### 3.2. Features Extraction

To train the various classifiers, we used the following four spectral features: PSD, MFCC, GTCC, and Wavelets. All four feature types are extracted for both RF and Audio datasets.

**Figure 2:**
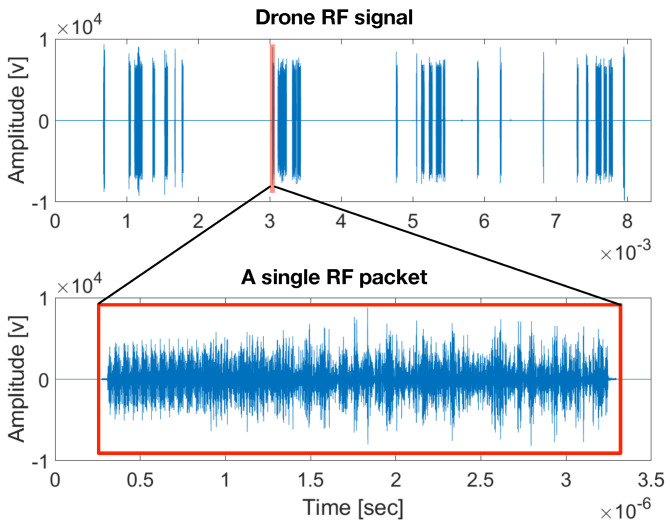
RF signal (from the DroneRF dataset).

The DroneRF and XBee datasets comprise sequential datapackets that include long silent segments, as depicted in Figure 2. Therefore, in the preprocessing stage, we first remove the silent intervals, leaving only the RF transmission packets. Each of the RF packets is composed of 4000 samples for the DroneRF dataset and 12,000 samples for the XBee dataset, while the audio packets include around 16,000 samples for the DroneAudio dataset.

#### 3.2.1. Power Spectrum Density 

The PSD is calculated as follows:

(1) $$X_{f}^{DFT}\left(k\right)=\sum_{j=1}^{N}\left[x_{t}\left(j\right)-\mathrm{Mean}\left(x_{t}\right)\right]W_{N}^{\left(j-1)(k-1\right)}$$

(2) $$X_{f}^{PSD}\left(k\right)=\frac{2}{f_{S}\cdot N}\cdot {\left|X_{f}^{DFT}\left(k\right)\right|}_{k\geq 0}^{2}$$

where $x_{t}$ denotes the input RF signal in the time domain, $f_{s}$ denotes the sampling frequency, *N* is the number of samples, and $W_{N}=e^{-2\pi i/N}$.

#### 3.2.2. MFCC and GTCC Cepstral Coefficients 

The extraction of the MFFC is depicted in Figure 1. First, the signal is transformed to the frequency domain using FFT, and then the power spectrum is computed. The power spectrum is resampled on the mel scale using a 16-band mel filter bank. Then, the log-energy of the mel-filter output is calculated. Finally, the MFCC is extracted by performing a Discrete Cosine Transform. The GTCC features are similarly extracted using GammaTone filters in place of the mel filters.

#### 3.2.3. Wavelets

Wavelet transforms are widely used as an efficient feature due to their time-frequency localization properties. In this work, we use the Wavelet Packet Decomposition (WPD) [45], which is a generalized form of the Discrete Wavelet Transform (DWT), for both RF and audio features extraction. While in the DWT, each tree-level is calculated by passing only the previous wavelet approximation coefficients through the discrete-time filters, in the WPD, more filters are used. Both the high-pass detail coefficients, and the low-pass (coarse) approximation coefficients are decomposed to create the full binary tree. We use 6 levels of decomposition producing 64 different sets of coefficients, and apply the continuous Meyer wavelet. Each leaf in the full binary tree represents a coefficient vector with a length equal to the number of input samples divided by 64. To reduce the number of coefficients, we suggest characterizing each vector with only two features: the Root Mean Square (RMS) and the Standard Deviation (STD) of each vector. Therefore, the wavelet-based feature vector is composed of 64·2=128 unique features.

### 3.3. Classification

**Figure 3:**
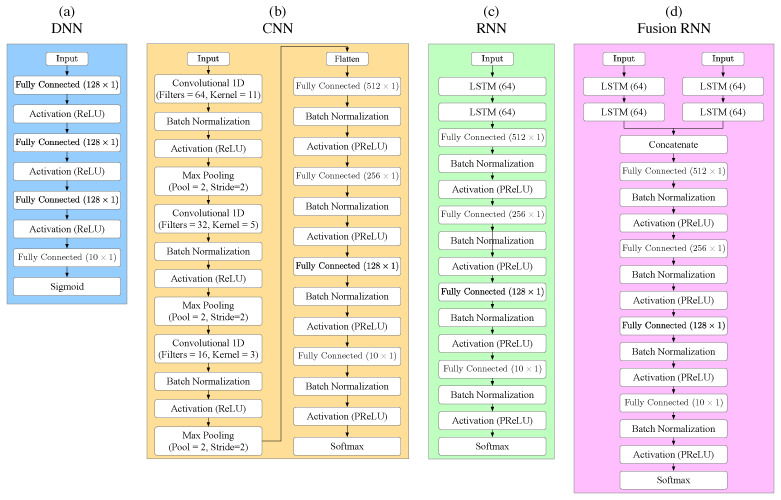
NN-based classifiers (**a**–**c**), and (**d**) the proposed fusion RNN-based network.

We examine four ML-based classification methods applied to drone detection: DNN, CNN, RNN and the classical SVM method for drone detection. Figure 3 depicts the three NN-based classifiers (DNN, CNN, and RNN), and the proposed fusion-RNN. The performance of the classifiers was evaluated for both RF and audio features. We also propose a fusion-RNN model, which requires the input of a concatenated feature vector that includes the MFCC/GTCC for both the RF input and the audio input. For the DNN classifier, all the extracted features (PSD-MFCC/GTCC-WPD) have been applied, while for the CNN, only raw data have been used. As an input vector for the RNN-based classifiers, we used the MFCC/GTCC and the WPD features. The classification performance is validated using a *K*-fold cross-validation technique [46] (with K=10) and evaluated using confusion matrices.

#### 3.3.1. DNN

The DNN classifier is composed of three hidden fully connected (FC) layers with 128 neurons at each layer, as depicted in Figure 3a. The rectified linear unit (ReLU) activation function is used for all three hidden layers, while the Sigmoid function is applied for the output layer. The number of neurons in the output FC layer represents the number of drone classes (i.e., ten categories). The DNN training is carried out using a back-propagation algorithm and ADAM optimizer to minimize the Mean Squared Error (MSE) loss function. The training process was conducted over 200 epochs with a batch size of 10.

#### 3.3.2. CNN

Figure 3b depicts the CNN used in this study, which is motivated by the LeNet architecture [47]. The CNN classifier consists of three convolution modules, three fully connected layers, and an output layer employed by a SoftMax function. Each convolution module is composed of a convolution layer, batch normalization, ReLU activation layer, and Max-pooling layer. The convolution layers consist of 64, 32, and 16 filters, and 11, 5, and 3 kernels size for the first, second and last layer, respectively. The three fully connected layers include 512, 256, and 128 neurons, respectively. Each FC layer is followed by a batch normalization and parametric ReLU activation. For the CNN, cross entropy is used as the loss function. The CNN training was conducted over 100 epochs with a batch size of 10.

#### 3.3.3. RNN

A recurrent neural network (RNN) is a type of neural network that contains memory elements and, hence, is well suited for analyzing sequential data. The LSTM is a standard RNN for which the simple neurons are replaced with unique LSTM memory modules that include several gates (i.e., input, forget, and output gates). Like the LSTM, the gated recurrent unit (GRU) includes a gating mechanism with only two gates (i.e., input and reset gate), resulting in fewer parameters. GRU’s performance of certain tasks was found to be similar to that of LSTM, and therefore, in this work, we examine which of the two gating mechanisms is better.

Figure 3c depicts the detailed RNN network (LSTM and GRU). Both LSTM and the GRU architectures were implemented with two LSTM/GRU layers, each containing a state vector of size of 64, followed by three fully connected hidden layers composed of 256, 128, and 10 neurons, respectively. The first two FC hidden layers are followed by a batch normalization and PReLU activation function, while in the last output layer, the SoftMax activation function is used. The cross-entropy is used as the loss function, and the training was conducted over 100 epochs with a batch size of 10.

#### 3.3.4. Support Vector Machine

Support Vector Machines (SVMs) are a type of supervised machine learning algorithm used for classification and regression tasks. Given labeled training data, the SVM algorithm finds an optimal hyperplane (i.e., a decision boundary) that separates data points into different classes. In two-dimensional space, this hyperplane is a line dividing a plane into two parts, where each class lies on either side. Multi-class classification is performed by constructing a hyperplane to divide data into multiple groups with maximum variance. We used the Radial Basis Function (RBF) as the tested SVM kernel decision function for classifying *K* classes using K(K−1)/2 kernels.

#### 3.3.5. Fusion RNN-Based Architecture

In this work, we propose a new approach using fusion of both RF signature and acoustic features for efficient and accurate drone classification. Figure 3 depicts the fusion model architecture based on two different RNN networks used for feature extraction. Each RNN network is implemented with two LSTM layers, each containing a state vector with a size of 64. The pre-trained RNN networks, previously used separately for each of the features, is used in the fusion model applying transfer learning. Since the LSTM slightly outperforms the GRU while using the RNN model, we implement only the LSTM layers in the fusion model.

The fusion of the two kinds of features is carried out by concatenating the two output vectors derived from the two second LSTM layers, thus generating a merged feature vector of size 128. The fusion model requires the input of a concatenated feature vector that includes either WPD or the MFCC/GTCC for both the RF input and the audio input.

The classification phase includes four fully connected hidden layers composed of 256, 128, and 10 neurons, respectively. The first two FC hidden layers are followed by a batch normalization and PReLU activation function, while in the last output layer, the SoftMax activation function is used. The cross-entropy is used as the loss function, and the training was conducted over 100 epochs with a batch size of 10. The main advantage of the proposed fusion approach is that it is immune to noise. Results show a significant improvement in accuracy in a noisy environment with low SNR levels.

## 4. Results

This section describes the experimental results for the various features used (PSD-MFCC/GTCC-WPD) and the four ML-based classification methods (DNN, CNN, RNN and the classical SVM), as well as the proposed fusion RNN-based model. The proposed method has been evaluated with the three datasets (XBee, Drone RF, and Drone Audio) described in Section 3.

As described in Section 3, the DNN classifier is composed of three hidden FC layers, with 128 neurons in each layer. The CNN classifier consists of three convolution modules, three fully connected layers (composed of 512, 256, and 128 neurons), and an output layer employed by a SoftMax function. Both LSTM and the GRU architectures were implemented with two LSTM/GRU layers, each containing a state vector of size of 64, followed by three fully connected hidden layers.

The classification performance is validated using a *K*-fold cross-validation technique (with K=10) and evaluated using confusion matrices. The *K*-fold cross-validation consists of splitting the dataset into *K* subsets; then, iteratively, some of them are used to learn the model, while the others are exploited to assess its performance. The performance measure is calculated by averaging the values reported by each *K*-fold sets.

The hyper-parameters that are associated with the Adam optimizer have been used as the default for the training process. A learning rate of 0.001 has been chosen to control the weights update rate. The Adam hyper-parameters $\beta_{1}$ and $\beta_{2}$, which determine the initial decay rates used when estimating the gradient moments, have been defined as 0.9 and 0.999, accordingly, while the epsilon value that ensures stability during training has been defined as $10^{-8}$.

The dataset is divided into 90% samples used for training, 10% used for validation in each iteration throughout the training process, and 10% used for testing with new unknown samples that were not used in the training process.

The results show the efficiency of our approach for multi-class drone detection, demonstrating good discrimination between the different RF sources with a success rate of around 98% for 10 classes. The audio features were inferior to the RF, demonstrating a success rate of around 80% for 10 classes. The proposed fusion RNN-based approach yields the best accuracy results for a noisy environment.

This section is organized as follows: Section 4.1 evaluates the proposed method for the two RF-based datasets (Drone RF, and XBee), which include ten different types of drones each (10 classes). Section 4.2 evaluates the proposed method for the Audio datasets (drone audio), which also includes ten different types of drones (10 classes). To evaluate the robustness of the proposed approach, the performance of the various classifiers has been examined in a noisy environment adding Gaussian noise in varying SNR levels (up to −20 dB).

Finally, Section 4.3 evaluates the proposed fusion RNN-based model compared to other drone classification methods, based on only one kind of feature, and emphasizes the advantages of this model, especially in a noisy environment.

### 4.1. Drone RF Datasets

Table 2 summarizes the classification results for the two RF-based datasets for the four feature types and the various classifiers. The best classification accuracy (for the Drone RF) is achieved while using LSTM with GTCC, demonstrating a success rate of 98.82%. The LSTM and GRU achieve similar accuracy and outperform both DNN and CNN, with an improvement of up to about 3% for the GTCC. The DNN shows better results compared to the CNN, demonstrating ∼2% and ∼7% improvement for the MFCC and PSD, respectively. The SVM shows better results than the CNN except for the PSD features. For the XBee dataset, similar results are achieved (over 99%) for all classifiers and the various features. Regarding all five of the classifiers, the MFCC and GTCC demonstrate better accuracy compared to the PSD and the WPD features, while the GTCC is slightly better. For example, the GTCC shows an improvement of 3.3% compared to WPD for the DNN classifier.

Our results have been compared to the results shown in five relevant references that used the same dataset [27,28,29,30,31] for different classifiers. The first three references utilize only the PSD features, while [31] uses both PSD and MFCC, and [30] uses continuous wavelet transform. Our approach outperforms all five references in terms of accuracy, demonstrating 90.2% compared to 87.4% for the CNN with PSD, 85.6% compared to 78.8% for the SVM with PSD, 93.9% compared to 90.3% for the SVM with MFCC. Moreover, the best results are achieved with our RNN-based classifiers (LSTM and GRU) showing an improvement of about 8% compared to the best published result out of all of the references used for comparison.

**Figure 4:**
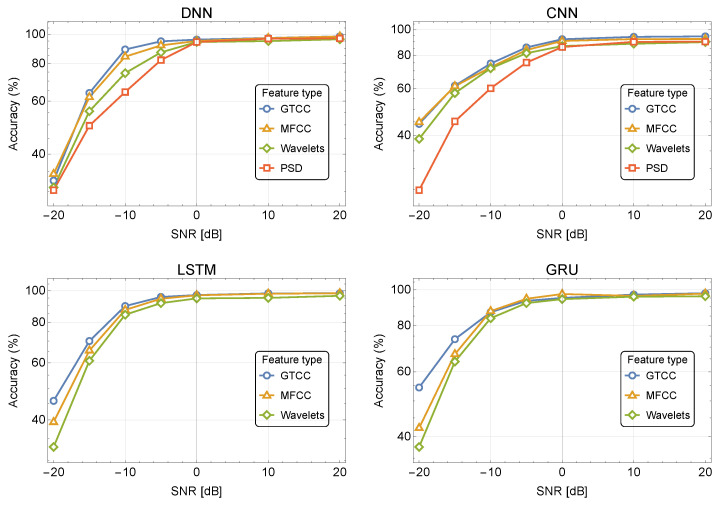
Accuracy as a function of SNR, for 10 classes using the DroneRF dataset in the presence of noise.

**Figure 5:**
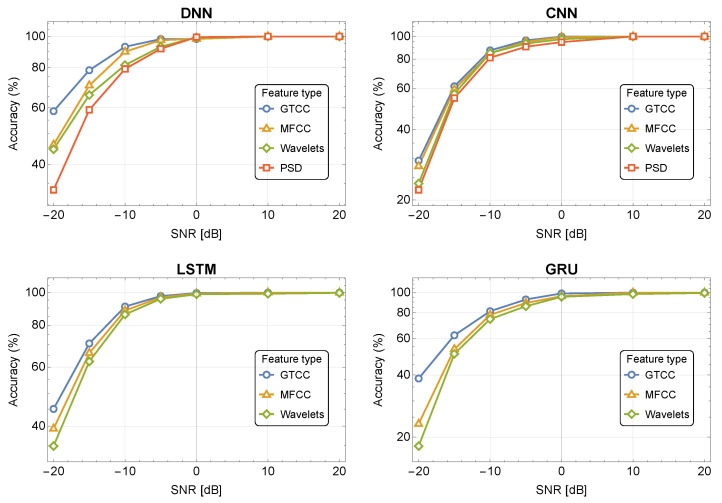
Accuracy as a function of SNR, for 10 classes using the XBee dataset in the presence of noise.

Figure 4 and Figure 5 depict the classification accuracy for different SNR values for the four NN-based classifiers using the DroneRF and XBee datasets, respectively. The results show that the GTCC and MFCC outperform the WPD and PSD, and are more immune to noise. For example, the DNN demonstrates an accuracy of 89.1%, 84.3%, 74.3%, and 64.3% for GTCC, MFCC, WPD, and PSD in noise of −10 dB SNR, respectively. The RNN-based classifiers (LSTM and GRU) demonstrate better noise immunity compared to the CNN and DNN while using MFCC/GTCC features.

### 4.2. Audio Datasets

Table 3 and Table 4 summarize the results for the Audio datasets with three-class and ten-class classifications, respectively, for the four feature types and the various classifiers. As expected, the results for the three-class scenario are significantly better compared to the more difficult case, which includes ten drones. The best three-class classification accuracy is achieved while using SVM with GTCC demonstrating a success rate of 99.5%. For all of the classifiers, the MFCC and GTCC show similar accuracy and demonstrate better accuracy compared to the PSD and the WPD features.

Our results have been compared to the results shown in seven relevant references that used the same dataset and the same features except the WPD (which is used only by us) for different classifiers, as shown in Table 3. For the three-class scenario, our approach shows comparable results to the existing work. The LSTM networks demonstrate around 98% accuracy and, in general, outperform the DNN and CNN for all of the features, including WPD.

Table 4 summarizes our results for the more interesting and complicated scenario (10-class). These particular results cannot be compared to existing work, due to the lack of a common multi-class audio dataset. As stated in Section 3.1.3, our unique 10-class drone audio dataset is composed of seven drones from [48], and three classes from [28]. The best 10-class classification accuracy is achieved while using DNN with GTCC, demonstrating a success rate of 79.9%. The MFCC and GTCC show similar accuracy and demonstrate better accuracy compared to the PSD and the WPD features.

**Figure 6:**
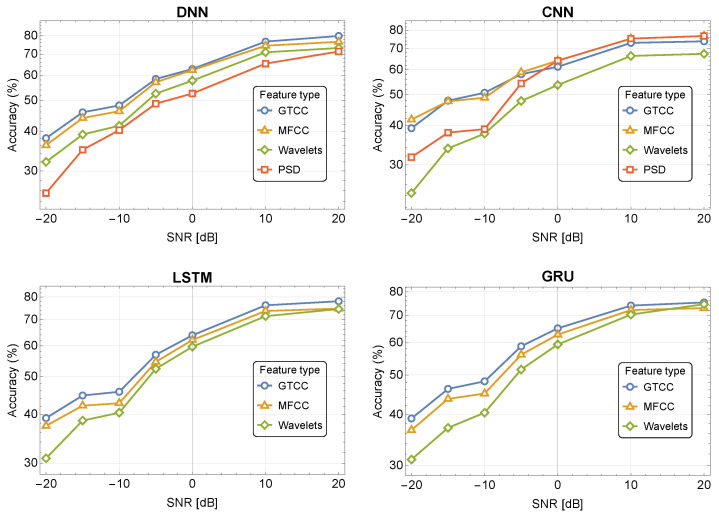
Accuracy as a function of SNR, for 10 classes using the DroneAudio dataset in the presence of noise.

Figure 6 depicts the 10-class classification accuracy for different SNR values for the four NN-based classifiers using our audio dataset. The results show that the GTCC and MFCC outperform the WPD and PSD and are more immune to noise. For example, the DNN demonstrates accuracy of 76.7%, 74.4%, 70.9%, and 65.3% for GTCC, MFCC, WPD and PSD in noise of 10 dB SNR, respectively. The CNN and DNN classifiers demonstrate better noise immunity compared to the LSTM for all of the features. The GRU shows the best noise immunity, especially for high noise levels while using GTCC and MFCC.

### 4.3. Fusion RNN-Based Architecture

**Figure 7:**
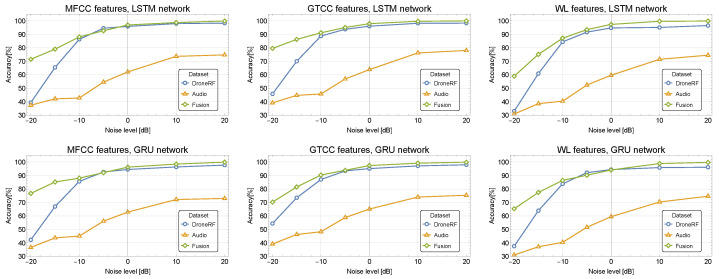
Classification accuracy for different SNR values for the fusion RNN-based model applied to the Audio and DroneRF datasets.

**Figure 8:**
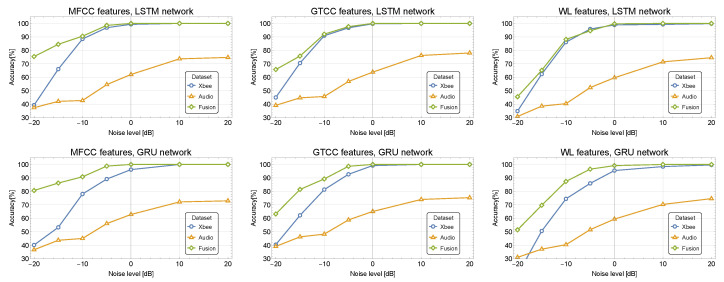
Classification accuracy for different SNR values for the fusion RNN-based model applied to the Audio and XBee datasets.

Figure 7 and Figure 8 depict the classification accuracy for different SNR values for the proposed fusion RNN-based model applied to the Audio 10-class dataset merged with DroneRF and XBee datasets, respectively. The classification performance is validated using a K-fold cross-validation technique (with K=10) and evaluated using confusion matrices [46].

Figure 7 shows that the GTCC and MFCC features outperform the WPD and are more immune to noise. For example, the LSTM demonstrates accuracy of 79.6%, 71.5% and 58.9% for GTCC, MFCC, and WPD for SNR of −20 dB, respectively. The GTCC outperforms the MFCC, especially in high noise levels, demonstrating an improvement of 11% at −20 dB compared to MFCC.

The main advantage of the proposed fusion approach is its noise immunity. The results show a significant improvement in accuracy in a noisy environment with low SNR levels. Using the LSTM classifier with MFCC for the Audio and DroneRF datasets leads to an improvement of 81% and 20% for SNR of −20 dB and −15 dB, respectively. Similarly, using the GRU with MFCC, an improvement of 82% and 27% is achieved for SNR of −20 dB and −15 dB, respectively.

Although for relatively low noise, good results and similar behavior are obtained for all of the features, the use of the proposed merging approach still yields an average improvement of 1.5% and 3.5% for the MFCC/GTCC and the WPD, respectively. Similar results and insights are achieved with the XBee dataset, demonstrating a better improvement in high noise levels for all of the features, as depicted in Figure 8.

## 5. Summary and Conclusions

This research proposes a new RNN-based approach using a fusion of radio frequency and acoustic features for supervised drone classification. The proposed method has been evaluated with common datasets (XBee, Drone RF, and Drone Audio), and compared against different ML-based classification methods (DNN, CNN, RNN, and the classical SVM) using various features (PSD-MFCC/GTCC-WPD).

Our results are compared to those shown in five relevant references using the same dataset for different classifiers. The proposed RNN-based classifiers (LSTM and GRU) outperform all five references in terms of accuracy, demonstrating an improvement of about 8% compared to the best published result.

As far as we know, this is the first time a fusion approach merging RF signatures and acoustic features has been used to improve drone classification. The main advantage of the proposed fusion approach is its noise immunity. Results show a significant improvement in accuracy in a noisy environment with low SNR levels. An improvement of about 80% is achieved while using the LSTM classifier for SNR of −20 dB.

## Figures and Tables

**Table 1:** Summary of the DroneAudio dataset.

Class	Drone	Packets
1	Custom-built UAV	3931
2	Phantom 4 PRO	4041
3	Phantom 4 GL300C	4584
4	enRoute Zion PG560	4066
5	YH-19HW	4395
6	Self-assembled UAV	3635
7	Intel Aero Ready-To-Fly	4437
8	Bebop	3305
9	Mambo	3305
10	Interference	3305

**Table 2:** Classification accuracy results [%] for the DroneRF and XBee datasets.

Work	Year	Dataset	Classifier	Features			
				PSD	MFCC	GTCC	Wavelets
[27]	2019	DroneRF	DNN	46.80			
[28]	2020	DroneRF	CNN	87.40			
[29]	2021	DroneRF	XGBoost	70.09			
[31]	2022	DroneRF	SVM	78.85	90.30		
[30]	2023	XBee	KNN				100
			DNN	97.10	95.40	95.50	92.20
			SVM	85.62	93.92	95.84	92.41
		DroneRF	CNN	90.20	93.12	94.65	90.69
			GRU		97.96	98.65	96.39
OUR	2023		LSTM		98.44	98.82	96.87
		XBee	DNN	100	99.60	99.10	99.89
			CNN	99.66	98.97	98.90	98.90
			GRU		99.57	99.36	99.93
			LSTM		99.23	99.10	99.89

**Table 3:** Classification accuracy results [%] for the DroneAudio datasets with 3 classes.

Work	Year	Classifier	Feature			
			PSD	MFCC	GTCC	Wavelets
[15]	2017	SVM		97.40		
[32]	2017	RNN		81		
		RNN	57.16			
[33]	2019	CNN	92.94			
		CRNN	92.22			
[34]	2019	SVM		96.70		
[35]	2019	CNN	90.10			
[36]	2021	SVM		98.70	99.50	
[37]	2022	DNN		98.52		
		CNN		98.60		
		LSTM		98.60		
		C-LSTM		98.11		
OUR	2023	DNN	87.70	94.87	93.33	93.84
		CNN	92.42	78.89	79.21	78.11
		GRU		98.29	97.95	96.30
		LSTM		98.10	98.12	97.19

**Table 4:** Classification accuracy [%] of the proposed method (DroneAudio dataset with 10 classes).

Classifier	Feature			
	PSD	MFCC	GTCC	WPD
DNN	71.56	76.87	79.91	73.43
CNN	76.88	78.73	74.12	68.22
GRU		73.51	75.67	74.61
LSTM		75.01	78.95	75.13

## Data Availability

Requests for data should be forwarded to the original data authors. DroneRF: Al-Sa’d et al. [27], Zigbee: Ashus et al. [30], DroneAudio: Al-Emadi et al. [33], and SPCUP19: Deleforge et al. [44].
